# Rosai-Dorfman disease with spine involvement: A case report

**DOI:** 10.1097/MD.0000000000028413

**Published:** 2022-02-25

**Authors:** Haoran Jiang, Jipeng Song, Wancheng Lin, Meng Yi, Mingtao Yao, Lixiang Ding

**Affiliations:** Department of Spine, Beijing Shijitan Hospital, Capital Medical University, No. 10 Tieyi Road, Yangfangdian, Haidian District, Beijing, People's Republic of China.

**Keywords:** Rosai-Dorfman disease, spinal surgery, spinous process

## Abstract

**Rationale:**

Rosai-Dorfman disease (RDD) is a rare, benign, self-limiting disease, also known as sinus histiocytosis with giant lymphadenopathy. Skeletal involvement is rare, and this isolated bone lesion usually occurs in adults with no other symptoms. It is estimated that 0.6% to 1% of RDD cases have isolated or complicated spinal lesions, which may occur in the bone, dura, and spinal parenchyma, but spinal RDD has no pathologic clinical or imaging features.

**Patient concerns:**

A 25-year-old woman presented with complaints of low back pain without obvious causes for a month.

**Diagnosis:**

RDD with spinal involvement.

**Interventions:**

Resection of the spinous process of the third lumbar spine was performed under epidural anesthesia.

**Outcomes:**

At the time of discharge, the patient had no problems with autonomous activities and reported no discomfort. We also followed up the patient at 12 and 36 months after surgery, and the patient reported no discomfort, inconvenience, and no recurrence of symptoms. Imaging examination 1 year after surgery showed no recurrence.

**Lesson:**

This case suggests that surgery for RDD with spinal involvement may not require internal fixation.

## 1 Introduction

Rosai-Dorfman disease (RDD) is a rare, benign, self-limiting disease, also known as sinus histiocytosis with giant lymphadenopathy, which was proposed by Rosai and Dorfman in 1969.^[1,2]^ The disease occurs mostly in children and young adults and is typically characterized by painless enlargement of bilateral cervical lymph nodes with fever, elevated neutrophils, accelerated erythrocyte rate, and hyperglobulinemia.^[3]^ In addition to affecting the lymph nodes, approximately 45% of cases involve at least 1 other node.^[4]^ The most common external nodes involved were the skin, soft tissue, and nasal and paranasal sinuses (16%). The eyes, orbit, and bones account for 11%, the salivary glands and central nervous system account for 7%, and less frequently, the mouth, kidneys, respiratory tract, liver, tonsils, breast, gastrointestinal tract, and heart. Lesions involving the bone are usually multifocal and are associated with lymphadenopathy and other organ diseases.^[5,6]^ Skeletal involvement is rare, and this isolated bone lesion usually occurs in adults with no other symptoms. It is estimated that 0.6% to 1% of RDD cases have isolated or complicated spinal lesions, which may occur in the bone, dura, and spinal parenchyma, but spinal RDD has no pathologic clinical or imaging features.^[7,8]^

## 2 Case presentation

A 25-year-old woman presented with a chief complaint of low back pain without obvious cause of 1 month duration. The patient was asymptomatic and had not received any prior treatment. The pain was aggravated during lumbar bending and relieved during back extension. There was no night or hip pain. The patient had no previous medical history. The patient had no personal or family history.

On physical examination, the patient did not show symptoms of fever. The patient entered the ward in a forced position with a distressed expression and walked with a staggered gait. There was no cervical mass or systemic superficial lymph node enlargement.

Laboratory examinations findings: Leukocyte cell count (7.94 × 10^9^/L), Lymphocyte percentage (19.5%) neutrophil percentage (76.5%), platelet count (401 × 10^9^/L) and erythrocyte sedimentation rate (64 mm/h); liver function test showed total protein (91.5 g/L), globulin (46.2 g/L); The immune results showed total serum complement (58.6 U/mL), immunoglobulin IgG (17,9 g/L), immunoglobulin IgA (4.1 g/L), complement C3 (1.2 g/L).

**Figure 1:**
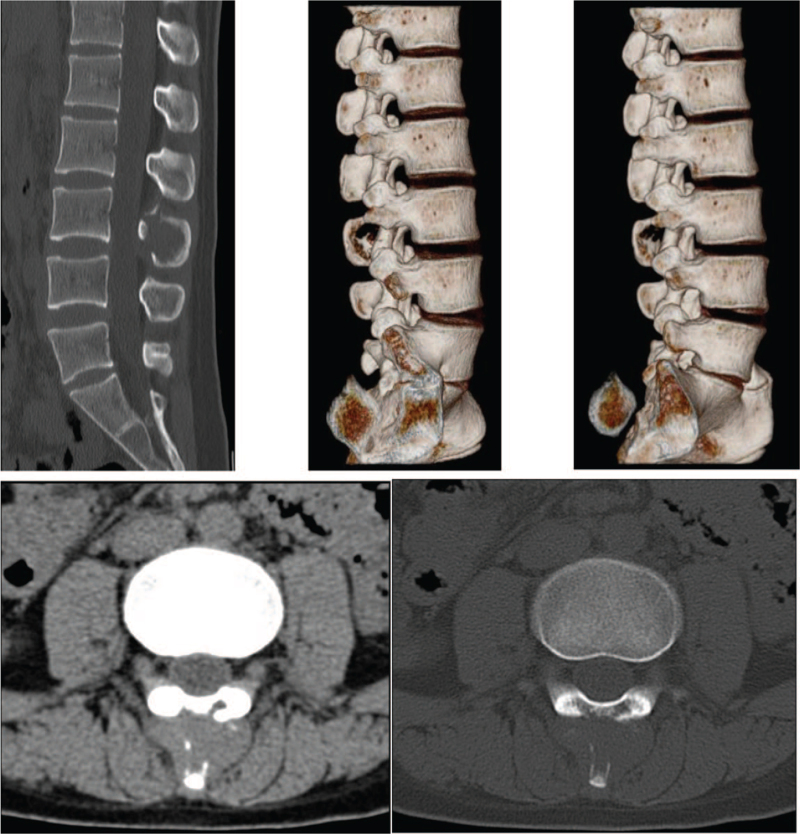
Preoperative CT images. The L3 pedicle and spinous process bone destruction, local bone cortex hours, soft tissue density shadow can be seen, CT value is 51HU, the boundary is unclear, and the range is approximately 1.9 cm × 1.9 cm.

**Figure 2:**
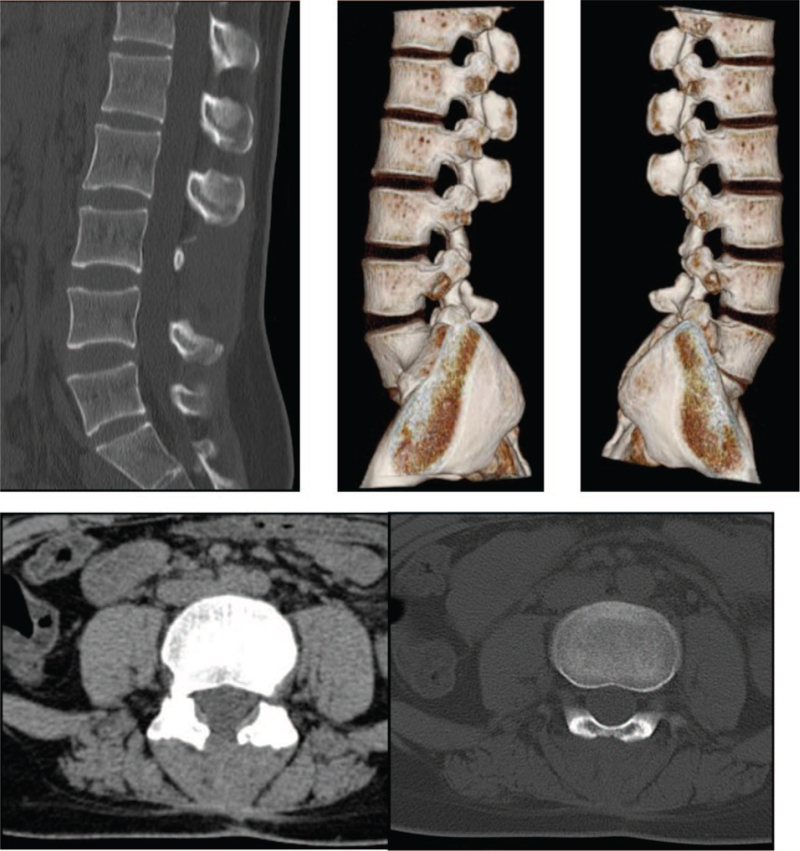
Preoperative MRI images & Postoperative MRI images. Preoperative: The normal bone in the right part of the L2 vertebral body, adjacent pedicle, and L3 accessory disappeared, and was partially replaced by abnormal mass-like and mass-like signals, such as slightly longer T1 and slightly longer T2. The lesion range of the L3 spinous process area was approximately 3.2 × 2.2 × 3.1 cm. Postoperative: The postoperative state of the lumbar spine showed absence of the L3 spinous process, good sequence, and curvature of the lumbar spine. The signals of the vertebral bodies and intervertebral discs are normal.

Imaging examination findings: Computed tomography (CT) revealed bone destruction in the third lumbar pedicle (Fig. 1). Lumbar magnetic resonance imaging (MRI) suggested that the right side of the second vertebral body, the pedicle of the adjacent vertebral body, and the adnexa of the third lumbar vertebral body were abnormal and enhanced (Fig. 2).

**Figure 3:**
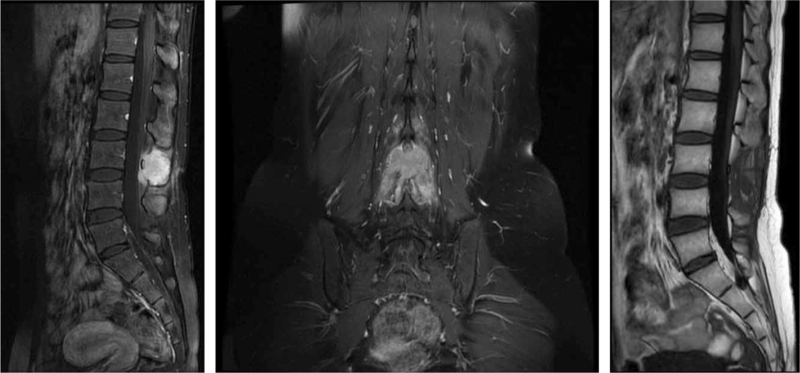
Staining results of pathological sections. Pathologic examination revealed a large number of foamy histiocytic cells and phagocytosis of lymphocytes, with the appearance of a large number of lymphocytes and neutrophils. Immunohistochemistry of the punctured tissue revealed that this tissue was positive for EMA, vimentin, LCA, CD68, CD79α, CD3, and S100, and negative for CK, CD1a, Ki-67, P53, and CD30.

We performed a spinous process puncture of the third lumbar vertebra under local anesthesia and pathological examination. Pathologic examination revealed a large number of foamy histiocytic cells and phagocytosis of lymphocytes, with the appearance of a large number of lymphocytes and neutrophils. Immunohistochemistry of the punctured tissue revealed that this tissue was positive for EMA, vimentin, LCA, CD68, CD79α, CD3, and S100, and negative for CK, CD1a, Ki-67, P53, and CD30 (Fig. 3). The final diagnosis of the patient was RDD with lumbar spine involvement.

No obvious surgical contraindications were observed. In this patient, only the spinous process was invaded, and the articular process and vertebral arch plate were intact. Therefore, in the preoperative discussion, we judged that the biomechanical properties of the patient could still be maintained after complete resection of the invaded spinous process. Resection of the spinous process of the third lumbar spine was performed under epidural anesthesia. During the surgery, we confirmed that the scope of RDD invasion was almost consistent with our preoperative judgment, so we did not perform internal fixation. The operation was without complications, with little bleeding, and the patient recovered well after the operation. At the time of discharge, the patient had no problems with autonomous activities and reported no discomfort.

**Figure 4:**
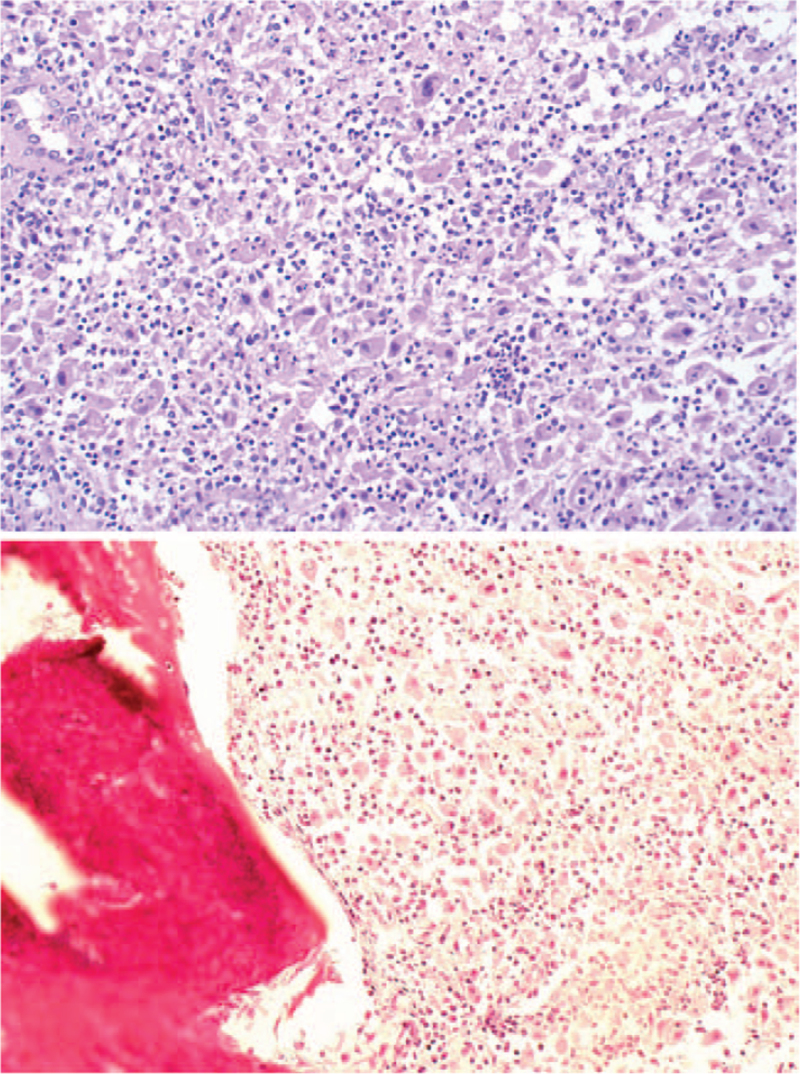
Postoperative CT images. The postoperative appearance of the L3 vertebral spinous process was irregular with a slightly high density and local subcutaneous strip shadow.

The patient was followed up 3 months after the operation, and the wound completely healed. The patient reported no discomfort at the surgical site and was able to move without support. We also followed up the patient at 12 and 36 months after surgery, and the patient reported no discomfort, inconvenience, and no recurrence of symptoms. Imaging examination 1 year after the surgery showed no recurrence (Figs. 2 and 4).

## 3 Discussion

RDD is a rare, benign disease that most commonly invades the cervical lymph node and affects extra-nodal sites, including the skin and brain, but is rare in bones. The bone injury is lytic, located inside the cancellous bone, and generally does not show expansion.^[8]^ RDD is often misdiagnosed as a tumor because its imaging findings are difficult to distinguish from tumors, especially when RDD invades uncommon sites. Generally speaking, the diagnosis of RDD requires the assistance of histopathology and cannot be confirmed solely by the patient's symptoms and imaging examination.^[9–11]^

The typical imaging characteristics of RDD on MRI are T1-weighted isosignals and T2-weighted mild hypersignals on magnetic resonance scans.^[1,3,8]^ On CT, the mass may present as isodense or high-density shadows. Most cases of bone involvement are single focal osteolytic lesions and flat bones, including the skull, vertebrae, and pelvis bones are mainly involved. Flat bone RDD has a clear osteolytic appearance with cortical destruction on radiographs. These lytic lesions are mostly aggressive and show cortical destruction and a periosteal reaction. It is worth mentioning that the vertebral body, which is the most commonly affected structure in the spine, shows imaging manifestations of vertebral collapse or vertebral flattening. Bone RDD presents intramedullary lytic lesions on CT and T2-weighted hyperintensity on MRI, possibly with reactive marrow edema.^[12]^

The specific histological features of RDD are positive S100 protein and CD68 immune markers, abnormal cell proliferation, and lymphocyte phagocytosis.^[13–15]^ The typical pathological manifestation of RDD is that the lymph node contour in the node is still retained, the lymph follicles are atrophic, and the germinal center is not obvious. The lymphatic sinus is highly dilated and filled with proliferative mononuclear or multinuclear histiocytes, accompanied by lymphocytes, plasma cells, and neutrophils. The morphology of the tissues and cells was consistent and well differentiated. The nucleus is large, vacuolar, round, or ovoid. The cytoplasm is rich and light red, and most of them can see intact lymphocytes, plasma cells, and neutrophils in the cytoplasm.

The uniqueness of this case is the simple invasion of the lumbar spinous process, which did not significantly affect the biomechanics of the spinal structure of this patient.^[16–18]^ For this reason, we did not perform internal fixation after the resection of the patient's tumor, which minimized the scale of the operation and reduced the patient's physical burden. The patient recovered to normal spinal activity within 3 months after surgery and showed no discomfort or recurrence at the 36-month follow-up.

Because RDD itself is rare, and cases involving the spine alone are rare, the initial diagnosis of this case was the result of several careful and detailed discussions, with a high degree of suspicion of RDD prior to the biopsy.

## 4 Conclusion

This case was characterized by isolated spinal involvement, and the patient's symptoms were low back pain without inducement. It is difficult to make a clear diagnosis based on the patient's symptoms and imaging examinations. Pathological examination requires an invasive operation to excise pathological tissues, which is why a detailed evaluation of the patient's condition is needed, and the diagnosis should be made as clear as possible. Therefore, it is challenging to confirm the diagnosis of isolated spinal RDD. At present, there are still many parts of RDD that have not been thoroughly studied, especially spinal RDDs. Therefore, careful diagnosis, surgery, and close follow-up are required for such patients.

## Author contributions

**Conceptualization:** Haoran Jiang, Lixiang Ding.

**Data curation:** Haoran Jiang, Lixiang Ding, Jipeng Song, Wancheng Lin, Meng Yi, Mingtao Yao.

**Formal analysis:** Haoran Jiang, Wancheng Lin.

**Investigation:** Lixiang Ding.

**Project administration:** Lixiang Ding.

**Resources:** Lixiang Ding, Jipeng Song.

**Supervision:** Lixiang Ding, Jipeng Song.

**Validation:** Lixiang Ding.

**Visualization:** Haoran Jiang, Lixiang Ding, Jipeng Song.

**Writing – original draft:** Haoran Jiang, Lixiang Ding, Jipeng Song, Wancheng Lin, Meng Yi, Mingtao Yao.

**Writing – review & editing:** Haoran Jiang, Lixiang Ding, Jipeng Song, Wancheng Lin.
